# The Magnitude of Black/Hispanic Disparity in COVID-19 Mortality Across United States Counties During the First Waves of the COVID-19 Pandemic

**DOI:** 10.3389/ijph.2021.1604004

**Published:** 2021-09-22

**Authors:** Cindy Im, Lalani L. Munasinghe, José M. Martínez, William Letsou, Farideh Bagherzadeh-Khiabani, Soudabeh Marin, Yutaka Yasui

**Affiliations:** ^1^ School of Public Health, University of Alberta, Edmonton, AB, Canada; ^2^ Technical University of Catalonia, Barcelona, Spain; ^3^ St. Jude Children’s Research Hospital, Memphis, TN, United States

**Keywords:** mortality, public health, COVID-19, SARS-CoV-2, race/ethnicity, health disparities

## Abstract

**Objectives:** To quantify the Black/Hispanic disparity in COVID-19 mortality in the United States (US).

**Methods:** COVID-19 deaths in all US counties nationwide were analyzed to estimate COVID-19 mortality rate ratios by county-level proportions of Black/Hispanic residents, using mixed-effects Poisson regression. Excess COVID-19 mortality counts, relative to predicted under a counterfactual scenario of no racial/ethnic disparity gradient, were estimated.

**Results:** County-level COVID-19 mortality rates increased monotonically with county-level proportions of Black and Hispanic residents, up to 5.4-fold (≥43% Black) and 11.6-fold (≥55% Hispanic) higher compared to counties with <5% Black and <15% Hispanic residents, respectively, controlling for county-level poverty, age, and urbanization level. Had this disparity gradient not existed, the US COVID-19 death count would have been 92.1% lower (177,672 fewer deaths), making the rate comparable to other high-income countries with substantially lower COVID-19 death counts.

**Conclusion:** During the first 8 months of the SARS-CoV-2 pandemic, the US experienced the highest number of COVID-19 deaths. This COVID-19 mortality burden is strongly associated with county-level racial/ethnic diversity, explaining most US COVID-19 deaths.

## Introduction

Coronavirus disease 2019 (COVID-19) suddenly emerged as a major cause of mortality in 2020 for human populations around the world. More deaths due to COVID-19 were reported during the first 8 months of the pandemic in the United States (US) than in any other country, with COVID-19-associated deaths approaching 200,000 as of September 2020 [1–3]. In the US, it is widely known that the burden of COVID-19 mortality fell disproportionately on US counties and states with higher proportions of Black and/or Hispanic residents during the first stages of the US COVID-19 public health crisis. This has been shown, for example, in separate analyses of counties of 10 large cities [4], all nonmetropolitan counties [5], and 28 states and New York City [6]. In the earliest stage of the COVID-19 pandemic (data collected as of April 2020), a nationwide county-level analysis found that US counties with higher proportions (≥13%) of Black residents had a 1.18-fold higher rate of COVID-19 death (95% CI: 1.00–1.40), adjusting for county-level characteristics such as age, poverty, comorbidities, and epidemic duration [7].

US counties are localized governmental units that coordinate the delivery of most social and public health services in the US; thus, US counties reflect not only the local economic, social, and political infrastructure, but the local scale of public health education and outreach [8]. The extent of US county-level Black/Hispanic COVID-19 mortality disparities before the broader rollout of improved patient care management measures and a long-term centralized public health response has not been comprehensively investigated. The purpose of this paper is as follows: 1) to quantify the gradient (dose-response relationship) of the disparity in COVID-19 mortality across all US counties nationwide with more granular categorizations of the proportions of non-Hispanic Black and Hispanic residents than those considered previously, over a time period that includes the first two US COVID-19 infection “waves” (January–September 2020); and 2) to estimate excess COVID-19 death counts and rates due to this disparity. An analysis of county-level COVID-19 mortality variation from the first waves of the US COVID-19 epidemic can provide insights into the preparedness of the US public health system to dynamically respond to a pandemic and can be utilized to inform the development and enhancement of equitable and effective local public health policy and actions.

## Methods

### Data Sources and Counties

Analyses included all 3,140 counties in the US Office of Management and Budget’s 2010 Standards for Delineating Metropolitan and Micropolitan Statistical Areas [9]. County-level population estimates for 2019 from the US Census Bureau [10] were used as denominators for COVID-19 death rates. The cumulative number of COVID-19 deaths from January 22 to September 15, 2020, by county (the numerators of the rates) were obtained from USAFacts [3], which aggregates COVID-19 deaths reported by the Centers for Disease Control and Prevention and state/local public health agencies. We chose September 15, 2020, as the data collection cutoff date because this cutoff 1) includes the first two major waves of COVID-19 infection in the US; 2) precedes the U.S. Food and Drug Administration (FDA) approval of the first antiviral drug for COVID-19 (remdesivir) (October 22, 2020); and 3) precedes the January 2021 US presidential executive orders mandating COVID-19 public health measures. An analysis of COVID-19 mortality from the first two waves of the US COVID-19 epidemic is less likely to include biases introduced by the availability of new COVID-19 treatments and a more centralized public health response. We linked county-level demographic data from the 2010 US Census [10]. County-level data on urbanization level of residence was obtained from the 2013 National Center for Health Statistics (NCHS) Urban-Rural Classification Scheme for Counties [11]. Given that all data used in this study is publicly accessible, participant informed consent and study approval by an institutional review board were not required.

### Statistical Analyses

Demographic characteristics, poverty level, and COVID-19 mortality descriptive statistics were calculated for the 3,140 counties. The percentages of residents in each county identifying as non-Hispanic Black (i.e., Black alone or in combination with other races) and as Hispanic (Spearman’s rank-order correlation between the two percentages was 0.4) were categorized into: percent Black <5%, 5–9%, 10–14%, 15–19%, 20–24%, 25–29%, 30–34%, 35–39%, 40–42%, and ≥43%; and percent Hispanic <15%, 15–19%, 20–24%, 25–29%, 30–34%, 35–39%, 40–44%, 45–49%, 50–54%, and ≥55%. The <5% Black and <15% Hispanic county reference groups were chosen *a priori* based on the county population distributions to create reference groups of roughly equal population size while rounding the racial/ethnic resident proportion to the nearest multiple of five (Table 1). Specifically, the sets of reference counties with <5% Black and <15% Hispanic residents corresponded to approximately 1 million county residents (∼1.1 million and ∼0.8 million, respectively). Percentages of county residents aged 60–69 years, 70–79 years, and ≥80 years were categorized into percent 60–69 years old <5.0%, 5.0–9.9%, and ≥10.0%; percent 70–79 years old <6.0%, 6.0–6.9%, and ≥7.0%; and percent ≥80 years old <4.0%, 4.0–4.9%, 5.0–5.9%, and ≥6.0%. The percentage of the county living under the federal poverty level was categorized into <10%, 10–14%, 15–19%, 20–24%, and ≥25%. The county urbanization level (urbanicity) was categorized by NCHS into six groups: large central metropolitan; large fringe metropolitan; medium metropolitan; small metropolitan; micropolitan; and noncore.

**TABLE 1:** Demographic characteristics, poverty level, and COVID-19 deaths of 3,140 US counties (United States, January 22 to September 15, 2020).

County characteristic	Number of counties		Population size in 1,000		Number of COVID-19 deaths	
	N	%	N	%	N	%
% Black residents						
<5	254	8.1	1,112	0.3	426	0.2
5–9	779	24.8	10,283	3.1	2,823	1.5
10–14	739	23.5	19,805	6.0	6,539	3.4
15–19	470	15.0	22,902	7.0	8,529	4.4
20–24	353	11.2	30,433	9.3	12,883	6.7
25–29	232	7.4	36,643	11.2	13,422	7.0
30–34	157	5.0	51,983	15.9	25,129	13.0
35–39	116	3.7	78,003	23.8	52,361	27.1
40–42	31	1.0	47,787	14.6	33,143	17.2
≥43	9	0.3	28,584	8.7	37,699	19.5
% Hispanic residents						
<15	33	1.0	81	<0.1	7	<0.1
15–19	153	4.9	1,659	0.5	269	0.1
20–24	528	16.8	9,460	2.9	2,439	1.3
25–29	690	22.0	18,003	5.4	6,207	3.2
30–34	647	20.6	35,981	11.0	13,553	7.0
35–39	537	17.1	68,626	21.0	31,329	16.2
40–44	356	11.3	93,588	28.6	55,913	29.0
45–49	156	5.0	92,288	28.2	69,359	36.0
50–54	30	1.0	7,446	2.3	13,418	7.0
≥55	10	0.3	396	0.1	460	0.2
% population living below poverty level						
<10	583	18.5	90,883	27.7	45,820	23.7
10–14	1,171	37.3	138,942	42.4	77,532	40.2
15–19	791	25.2	69,090	21.1	42,999	22.3
20–24	376	12.0	20,988	6.4	14,559	7.5
≥25	219	7.0	7,628	2.3	12,044	6.2
% residents aged 60–69 years						
<5	780	24.8	42,561	13.0	12,505	6.5
5–9	1,774	56.5	226,621	69.2	113,968	59.1
≥10	586	18.7	58,350	17.8	66,481	34.5
% residents aged 70–79 years						
<6	806	25.7	45,913	14.0	12,692	6.6
6-<7	1,057	33.6	99,381	30.3	39,318	20.4
≥7	1,277	40.7	182,238	55.6	140,944	73.0
% residents aged ≥80 years						
<4	432	13.8	13,920	4.2	3,536	1.8
4-<5	990	31.5	68,345	20.9	23,367	12.1
5-<6	1,084	34.5	116,384	35.5	52,180	27.0
≥6	634	20.2	128,883	39.3	113,871	59.0
Urbanicity						
Large central metro	67	2.1	100,299	30.6	79,119	41.0
Large fringe metro	368	11.7	82,475	25.2	52,606	27.3
Medium metro	372	11.9	68,841	21.0	32,584	16.9
Small metro	357	11.4	29,853	9.1	11,293	5.9
Micropolitan	641	20.4	27,294	8.3	10,103	5.2
Noncore	1,335	42.5	18,768	5.7	7,249	3.8

Abbreviations: %, percent.

County-level Poisson regression with independent Gaussian random intercepts for individual counties was used to evaluate the associations between the county percentages of Black and Hispanic residents and the rate of COVID-19 deaths, adjusting for county-level older-age resident percentages, poverty level, and urbanicity, where the logarithm of the 2019 county-level population sizes was used as an offset. The target parameters of interest were adjusted rate ratios of COVID-19 death in association with higher county-level percentages of Black and Hispanic residents, relative to the counties with <5% Black and <15% Hispanic residents. Since the reference counties with <5% Black and <15% Hispanic residents were all rural counties with population sizes <10,000, we repeated the Poisson regression analysis, adjusting for the same or comparable variables, among rural counties (defined as micropolitan and noncore by the NCHS Urban-Rural Classification Scheme) with the population size <10,000 in order to assess the robustness of our disparity gradient estimates.

The excess number of COVID-19 deaths in each county was estimated by subtracting the county’s “predicted” number of COVID-19 deaths, obtained from the Poisson model-fitted COVID-19 death rates using each county’s predictor characteristics, from the “predicted under no disparity” COVID-19 death count. The “predicted under no disparity” COVID-19 death count considered a counterfactual situation of no racial/ethnic disparity, i.e., all adjusted rate ratios of COVID-19 death in associations with county-level percentages of Black and Hispanic residents were forced to the null value of 1.0 in the fitted model, while all the other predictor effects remained as estimated [12]. The excess COVID-19 deaths are presented in two ways: a table of excess deaths due to COVID-19 by county-level percentages of Black and Hispanic residents to express the total burden of the disparity and a map of counties with 50 or more excess COVID-19 deaths per 100,000 population (approximately corresponding to the 80th percentile of excess COVID-19 mortality rates in the nation).

All statistical analyses were performed using Stata, version 15.0 (Stata Corp., College Station, TX, United States). RStudio version 1.3 (PBC, Boston, MA, United States) and R package “usmap” [13] were used to create the excess county deaths map. All statistical tests were two-sided.

## Results

Table 1 shows the demographic characteristics of the 3,140 US counties. A total of 192,954 COVID-19 deaths were reported in the US between January 22 and September 15, 2020. Counties with population sizes ranging from <10,000 to ≥3 million reported COVID-19 deaths (Supplementary Table S1); 571 counties did not report any COVID-19 deaths. The crude mortality rates and adjusted COVID-19 mortality rate ratios estimated by the Poisson model are shown in Table 2. Relative to counties with <5% Black residents, counties that were 5–9%, 10–14%, 15–19%, 20–24%, 25–29%, 30–34%, 35–39%, 40–42%, and ≥43% Black had 1.4, 1.8, 1.9, 2.0, 2.0, 2.3, 3.1, 3.1, and 5.4-fold higher rates of COVID-19 deaths, respectively, controlling for county-level poverty, proportion of older-age residents, urbanicity, and percentage of Hispanic residents. Similarly, relative to counties with <15% Hispanic residents, counties that were 15–19%, 20–24%, 25–29%, 30–34%, 35–39%, 40–44%, 45–49%, 50–54%, and ≥55% Hispanic had 1.6, 2.6, 3.5, 4.2, 5.1, 5.2, 5.0, 6.3, and 11.6-fold higher rates of COVID-19 deaths, respectively, controlling for county-level poverty, proportions of older-age residents, urbanicity, and percentages of Black residents. Among rural counties with population sizes <10,000 only (N = 654), we observed very similar gradients of racial/ethnic disparity in COVID-19 mortality rate ratios as those observed in the nationwide analysis (Supplementary Table S2).

**TABLE 2:** Crude mortality rates and adjusted COVID-19 mortality rate ratios by US county-level race/ethnicity, poverty level, age, and urbanization (United States, January 22 to September 15, 2020).

	Crude COVID-19 mortality rate (per 100,000 residents)	Adjusted COVID-19 mortality rate ratio		
		Estimate	95% CI	*p*-value
% Black				
<5	38.3	Reference		
5–9	27.5	1.40	1.11–1.78	0.005
10–14	33.0	1.78	1.41–2.26	<0.001
15–19	37.2	1.93	1.50–2.47	<0.001
20–24	42.3	2.05	1.58–2.65	<0.001
25–29	36.6	1.97	1.50–2.60	<0.001
30–34	48.3	2.27	1.69–3.03	<0.001
35–39	67.1	3.12	2.26–4.30	<0.001
40–42	69.4	3.12	1.97–4.96	<0.001
≥43	131.9	5.44	2.66–11.13	<0.001
% Hispanic				
<15	8.6	Reference		
15–19	16.2	1.60	0.59–4.28	0.35
20–24	25.8	2.63	1.00–6.92	0.051
25–29	34.5	3.49	1.33–9.17	0.011
30–34	37.7	4.15	1.58–10.94	0.004
35–39	45.7	5.07	1.92–13.37	0.001
40–44	59.7	5.23	1.98–13.82	0.001
45–49	75.2	4.98	1.87–13.25	0.001
50–54	180.2	6.30	2.24–17.77	0.001
≥55	116.0	11.55	3.64–36.64	<0.001
% under poverty				
<10	50.4	Reference		
10–14	55.8	1.25	1.11–1.41	<0.001
15–19	62.2	1.61	1.41–1.84	<0.001
20–24	69.4	2.51	2.14–2.93	<0.001
≥25	158.0	4.40	3.66–5.30	<0.001
% aged 60–69 years				
<5	29.4	Reference		
5–9	50.3	1.15	1.04–1.28	0.005
≥10	113.9	1.28	1.12–1.47	<0.001
% aged 70–79 years				
<6	27.6	Reference		
6-<7	39.6	1.05	0.94–1.17	0.39
≥7	77.3	1.19	1.06–1.34	0.003
% aged ≥80 years				
<4	25.4	Reference		
4-<5	34.2	1.09	0.95–1.24	0.23
5-<6	44.8	1.17	1.02–1.34	0.028
≥6	88.4	1.38	1.17–1.62	<0.001
Urbanicity				
Large central metro	78.9	Reference		
Large fringe metro	63.8	1.60	1.17–2.17	0.003
Medium metro	47.3	1.10	0.81–1.50	0.54
Small metro	37.8	1.11	0.80–1.54	0.51
Micropolitan	37.0	0.96	0.70–1.33	0.82
Noncore	38.6	1.07	0.77–1.47	0.69

Abbreviations: %, percent.

**FIGURE 1:**
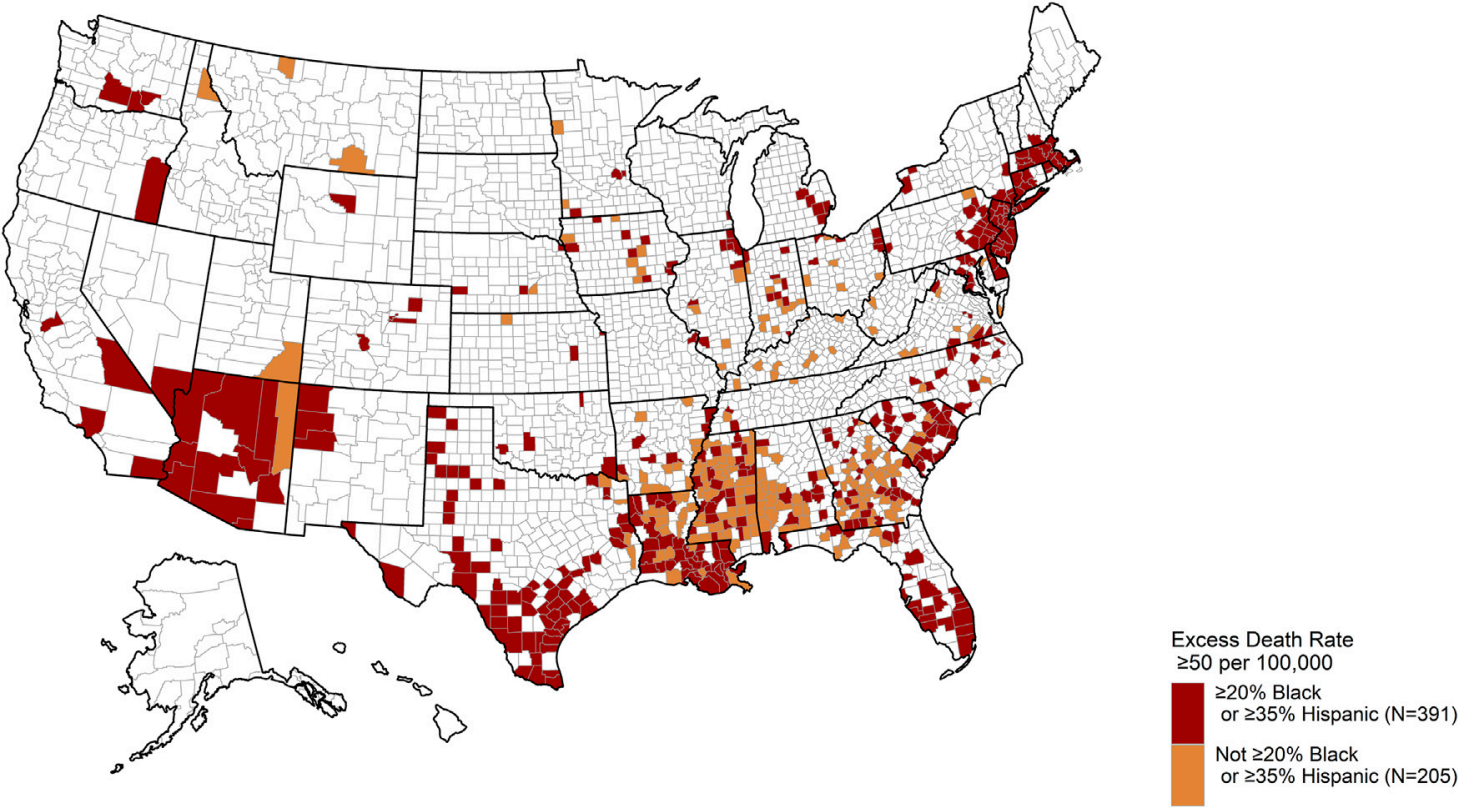
US county map highlighting counties with high excess COVID-19 death rates, stratified by counties with higher versus lower percentages of Black/Hispanic residents. All counties reporting a high excess COVID-19 mortality rate (≥50 deaths per 100,000 population) are shown in dark red or orange. Dark red counties represent counties with ≥20% Black or ≥35% Hispanic residents, while orange counties represent counties with <20% Black and <35% Hispanic residents (United States, January 22 to September 15, 2020).

Estimated excess COVID-19 deaths are shown in Table 3 by the percentages of Black and Hispanic county residents. Out of the 192,954 COVID-19 deaths observed during January 22 and September 15, 2020, 177,672 (or 92.1%) were excess compared to the counties that were <5% Black and <15% Hispanic. A US state map highlighting the counties with high (≥50) excess COVID-19 deaths per 100,000 population in the 8-month period is provided in Figure 1, with color coding distinguishing more racially/ethnically-diverse counties (defined by ≥20% Black or ≥35% Hispanic residents) from less diverse counties (defined by <20% Black and <35% Hispanic residents). The more racially/ethnically-diverse counties with high excess COVID-19 deaths were concentrated in Southern states with higher proportions of Black residents than the rest of the country (Louisiana, Georgia, North Carolina, and South Carolina), Southwestern states with large numbers of Hispanic residents (California, Texas, Arizona, New Mexico, and Florida), and the Northeastern states (New York, New Jersey, Pennsylvania, and Massachusetts).

**TABLE 3:** Excess numbers of COVID-19 deaths^a^ by percentages of Black and Hispanic residents in the county (United States, January 22 to September 15, 2020).

	% Hispanic										
% Black		<15	15–19	20–24	25–29	30–34	35–39	40–44	45–49	50–54	≥55
	<5	Reference	3 (20.0%)	21 (56.8%)	36 (66.7%)	21 (67.7%)	14 (63.6%)	21 (65.6%)	16 (66.7%)	8 (66.7%)	174 (89.2%)
	5–9	0 (0.0)^b^	48 (48.5%)	352 (66.0%)	561 (74.1%)	352 (77.0%)	265 (80.1%)	182 (77.8%)	126 (77.8%)	193 (86.2%)	22 (88.0%)
	10–14	0 (0.0)^b^	80 (56.6%)	830 (74.0%)	1,330 (79.8%)	1,081 (83.3%)	849 (86.4%)	586 (85.8%)	279 (85.6%)	74 (91.4%)	228 (95.0%)
	15–19	—	6 (75.0%)	361 (76.2%)	1,610 (82.1%)	2,140 (85.9%)	1,416 (88.3%)	1,285 (88.5%)	466 (87.4%)	7 (100.0%)	—
	20–24	—	2 (40.0%)	169 (78.2%)	1,093 (84.0%)	3,186 (86.6%)	2,625 (89.0%)	1,517 (90.2%)	483 (90.1%)	2,317 (92.1%)	—
	25–29	—	2 (66.7%)	44 (75.9%)	258 (84.9%)	2,973 (86.7%)	5,397 (89.1%)	2,051 (90.0%)	1,164 (89.8%)	—	—
	30–34	—	—	—	141 (86.0%)	1,791 (88.7%)	9,068 (91.0%)	7,682 (91.3%)	4,158 (91.0%)	—	—
	35–39	—	—	—	—	139 (91.4%)	8,496 (93.5%)	22,152 (93.8%)	15,920 (93.5%)	2,338 (94.8%)	—
	40–44	—	—	—	—	—	313 (93.4%)	14,457 (93.8%)	13,302 (93.6%)	3,014 (94.9%)	—
	≥45	—	—	—	—	—	—	2,027 (96.5%)	29,525 (96.3%)	4,791 (97.1%)	—

a Total excess deaths = 177,672 (92.1%) of 192,954.

b Excess deaths were rounded off to the least whole number thus providing zero excess deaths for cells with <1 excess death.

Abbreviations: %, percent; -, no COVID-19 death reported.

Each cell reports the number of excess COVID-19 deaths, and in parentheses, the fraction of observed COVID-19 deaths that are in excess, in counties with the specified % Black and % Hispanic resident proportions.

## Discussion

Our nationwide analysis of all 3,140 US counties found a steep, monotonically-increasing gradient (dose-response relationship) of adjusted COVID-19 mortality rates by increasing county-level percentages of Black and Hispanic residents during the first two waves of the US COVID-19 epidemic. During this time period, rates of COVID-19 deaths in counties with the highest percentages of Black and Hispanic residents were over 5-fold and 11-fold compared to counties with the lowest percentages of Black and Hispanic residents, respectively, after controlling for county-level poverty, the proportion of older-age residents, and urbanicity. Although the reference counties with <5% Black or <15% Hispanic residents consisted entirely of small rural counties, a secondary analysis of all rural counties with population sizes <10,000 (N = 654) showed very similar results (Supplementary Table S2), suggesting the county-level Black/Hispanic disparity gradient for COVID-19 deaths observed in the nationwide analysis is not due to any special characteristics of the reference counties; similar Black/Hispanic disparity gradients exist even among the smaller rural counties of the US.

It is clear that the magnitude of excess COVID-19 deaths in racially/ethnically diverse US counties during the first waves of the US COVID-19 epidemic was extraordinary. Of the 192,954 COVID-19 deaths in the US observed in the 8-month period, over 92% can be attributed to the county Black/Hispanic disparity gradient. Excess COVID-19 death rates of ≥50 excess deaths per 100,000 population over the 8-month pandemic period were observed in 596 counties, accounting for approximately 19% of US counties. Notably, 50 deaths per 100,000 population in an 8-month period is more than twice the crude death rate due to breast cancer among women in the US in a single year (25.5 deaths per 100,000 population) [14] and over one-quarter of the annual crude death rate due to diseases of the heart (198.8 deaths per 100,000 population) or cancers of all types (183.9 deaths per 100,000 population), the two leading causes of death in the US population [15]. Had the observed Black/Hispanic disparity gradient been absent, the rate of COVID-19 deaths in the US would have been 4.7 deaths per 100,000 population (92% lower). This hypothetical rate of COVID-19 mortality under no disparity is comparable to larger Organisation for Economic Co-operation and Development (OECD) countries with more moderate COVID-19 mortality rates during the same period such as Finland (6.5 deaths per 100,000 population) and Norway (5.3 deaths per 100,000 population), but still substantially higher than OECD countries with low mortality rates such as Australia (3.6 deaths per 100,000 population), Japan (1.4 deaths per 100,000 population), South Korea (0.9 deaths per 100,000 population), and New Zealand (0.5 deaths per 100,000 population) [2].

Characterizing the disparity in COVID-19 mortality by geographically localized, county-level variations in Black/Hispanic diversity during the first waves of the US COVID-19 public health crisis is critically important. Our results suggest the US public health system was poorly equipped to mitigate the racial/ethnic health disparities of the initial phase of the COVID-19 crisis at a localized level. They are also consistent with, but show a greater extent of disparity than, numerous reports of Black/Hispanic disparities in COVID-19 infections, morbidity, and mortality, all of which were largely limited to analyses of data from the first wave of US COVID-19 infections [4–7, 16–20]. While this analysis cannot show or adjust for specific individual-level risk factors that contribute to county-level racial/ethnic disparities in COVID-19 mortality, there are many possible underlying causes. The most common explanatory factors that have been described in the literature include factors related to the social and structural determinants of health, i.e., longstanding inequities in socioeconomic status, education and employment opportunities, adequate housing, and affordable and timely healthcare access, and the disproportionate risk of underlying comorbidities in racial/ethnic minority populations [21]. These factors not only reduce the means by which Black/Hispanic individuals can engage in public health measures that prevent COVID-19 infection (e.g., social distancing; sheltering-in-place) but may also result in poorer health outcomes after infection [21]. Specifically, recent research has shown that Black and Hispanic individuals are more likely to live and/or be employed in environments with higher risks of infection (e.g., overcrowded housing, service occupations requiring face-to-face contact) [7, 18, 19], but they are also less likely to seek COVID-19 testing and necessary care while sick [17, 21]. A previous report describing Hispanic/Latino county-level COVID-19 infection and mortality rate disparities has further hypothesized causes more specific to Hispanic/Latino populations: occupational exposures related to employment in food industries deemed “essential” (e.g., meatpacking plants), and decreased access to timely healthcare due to language and immigration documentation barriers [18].

### Limitations

A limitation of our study is that potential errors in COVID-19-associated death reporting by local and national public health agencies cannot be excluded. Not adjusting for other county-level potential confounding factors—including county-level indicators of public health services delivery efficacy (e.g., county-level comorbidity rates), insurance coverage, environmental exposures (e.g., air pollutant levels), and household occupancy levels—may also be considered a limitation. Arguably, however, county-level racial/ethnic disparity in COVID-19 mortality must include the county-level racial/ethnic disparities in these aforementioned county risk factors; therefore, our quantification of COVID-19 mortality disparity should not “adjust out” these potentially causal factors. An earlier analysis showed many of these county-level risk factors had weak, non-significant associations with county-level COVID-19 mortality rates [7]. Because historical disparities in health and healthcare access continue to persist among Black and Hispanic Americans [22], our analysis focused on mortality disparities by county-level variations in Black and Hispanic residents; future analyses including other vulnerable racial/ethnic minority groups may provide additional insights. Lastly, the lack of detailed individual-level data to quantify the extent to which individual-level characteristics impact COVID-19 mortality rates is another important limitation. Our analysis only quantified the gradient of racial/ethnic disparity in county COVID-19 mortality rates and associated excess deaths: the causes of the steep disparity are not identifiable in our study.

### Conclusion

It is an important first step to quantify and recognize the extent of racial/ethnic disparities in health outcomes across US counties during times of public health crisis in order to make public health policies and actions to address them. The primary strength of our study is that it is a population-based study including all US counties that quantified the disparity gradient and excess COVID-19 deaths by county-level Black and Hispanic diversity during the first two waves of the US COVID-19 epidemic. We found that COVID-19 mortality risk varied from county to county in a strict dose-response fashion with county racial/ethnic diversity, which is critically useful information that can be utilized for determining future public health policy and action. Specifically, we observed steep gradients in COVID-19 mortality rates across US counties by county-level percentages of Black and Hispanic residents. The absence of these racial/ethnic gradients would have corresponded to a 92% lower count of COVID-19 deaths nationwide during this pandemic period, making the crude US COVID-19 mortality rate similar to Finland and Norway, two high-income countries with far fewer COVID-19 mortalities during the same time period. While the causes of this community-level racial/ethnic disparity in COVID-19 mortality in the US are numerous and complex, our results corroborate previous research that suggests the US public health system was poorly equipped to mitigate racial/ethnic health disparities during the initial stages of the pandemic crisis [23]. Public health policies for implementing an initial pandemic public health response must consider actions and strategies that tackle racial/ethnic disparities in infectious disease-related morbidity/mortality.
