# Corrigendum: The Magnitude of Black/Hispanic Disparity in COVID-19 Mortality Across United States Counties During the First Waves of the COVID-19 Pandemic

**DOI:** 10.3389/ijph.2021.1604486

**Published:** 2021-12-20

**Authors:** Cindy Im, Lalani L. Munasinghe, José M. Martínez, William Letsou, Farideh Bagherzadeh-Khiabani, Soudabeh Marin, Yutaka Yasui

**Affiliations:** ^1^ School of Public Health, University of Alberta, Edmonton, AB, Canada; ^2^ Technical University of Catalonia, Barcelona, Spain; ^3^ St. Jude Children’s Research Hospital, Memphis, TN, United States

**Keywords:** mortality, public health, COVID-19, SARS-CoV-2, race/ethnicity, health disparities

In the published article there was an error regarding the attribution of author Lalani L. Munasinghe*.* This author shares first authorship, and contributed equally to the final publication as the first named author.

The authors apologize for this error and state that this does not change the scientific conclusions of the article in any way. The original article has been updated.

