# Supplementary material for: The Magnitude of Black/Hispanic Disparity in COVID-19 Mortality Across United States Counties During the First Waves of the COVID-19 Pandemic
Source: Int J Public Health. 2021 Sep 22;66:1604004. doi: 10.3389/ijph.2021.1604004 (PMC8493931; doi:10.3389/ijph.2021.1604004)
Supplement: Supplementary file 1 [file DataSheet1.PDF]

International Journal of Public Health

Title: The Magnitude of Black/Hispanic Disparity in COVID-19 Mortality across United States Counties during the First Waves of the COVID-19 Pandemic

SUPPLEMENTARY MATERIAL

Supplementary Tables

Table S1: COVID-19 deaths by county population size for 3,140 US counties (United States, January 22 to September 15, 2020)

County characteristic	Number of counties		Population size in 1,000		Number of COVID-19 deaths	
	N	%	N	%	N	%
County population size						
<10,000	717	22.8%	3,892	1.2%	1,390	0.7%
10,000-19,999	603	19.2%	8,866	2.7%	3,432	1.8%
20,000-29,999	389	12.4%	9,556	2.9%	4,029	2.1%
30,000-39,999	249	7.9%	8,670	2.7%	2,947	1.5%
40,000-49,999	193	6.2%	8,591	2.6%	3,435	1.8%
50,000-99,999	391	12.5%	27,493	8.4%	9,566	5.0%
100,000-149,999	155	4.9%	18,862	5.8%	6,798	3.5%
150,000-199,999	108	3.4%	18,486	5.6%	6,878	3.6%
200,000-299,999	105	3.3%	25,623	7.8%	10,111	5.2%
300,000-499,000	91	2.9%	35,364	10.8%	19,743	10.2%
500,000-999,999	94	3.0%	68,044	20.8%	49,959	25.9%
1,000,000-1,499,999	21	0.7%	26,059	8.0%	19,097	9.9%
1,500,000-1,999,999	8	0.3%	13,677	4.2%	12,313	6.4%
2,000,000-2,499,999	7	0.2%	15,530	4.7%	13,108	6.8%
2,500,000-2,999,999	3	0.1%	7,912	2.4%	11,279	5.8%
≥3,000,000	6	0.2%	30,902	9.4%	18,869	9.8%

Table S2: Adjusted COVID-19 mortality rate ratios for the subset of rural counties with N<10,000 (United States, January 22 to September 15, 2020)

	Number of counties (N = 654)	Adjusted COVID-19 mortality rate ratio[a]		
		Estimate	95% CI	P-value
% Black				
<5	223	Reference		
5-9	318	1.08	0.76-1.52	0.67
10-14	95	2.59	1.70-3.95	<0.001
≥15	18	4.18	2.09-8.35	<0.001
% Hispanic				
<15	32	Reference		
15-19	85	1.48	0.47-4.63	0.50
20-24	155	2.08	0.69-6.28	0.19
25-29	153	2.24	0.75-6.73	0.15
30-34	87	3.27	1.06-10.04	0.039
35-39	57	3.47	1.09-11.06	0.035
40-44	40	6.20	1.94-19.77	0.002
45-49	29	5.39	1.63-17.80	0.006
50-54	10	4.33	1.00-18.82	0.051
≥55	6	11.90	2.77-51.09	0.001
% under poverty				
<10	83	Reference		
10-14	299	1.36	0.81-2.28	0.24
15-19	132	1.64	0.93-2.90	0.090
20-24	79	3.12	1.70-5.71	<0.001
≥25	61	6.49	3.52-11.96	<0.001
% aged 60-69 years				
<5	182	Reference		
5-9	267	1.20	1.04-1.28	0.32
≥10	205	1.16	1.11-1.47	0.47
% aged 70-79 years				
<6	185	Reference		
6-<7	151	0.90	0.61-1.33	0.60
≥7	318	1.00	0.69-1.45	1.00
% aged ≥80 years				
<4	103	Reference		
4-<5	167	0.94	0.60-1.46	0.78
5-<6	161	1.03	0.65-1.64	0.88
≥6	223	1.25	0.79-1.97	0.34
Urbanicity				
Micropolitan	51	Reference		
Noncore	603	0.77	0.46-1.27	0.30

[a]Rate ratios were obtained from a mixed-effects Poisson regression model and adjusted for all covariates shown in the table.
